# Functional Characterization of RNA Silencing Suppressor P0 from Pea Mild Chlorosis Virus

**DOI:** 10.3390/ijms21197136

**Published:** 2020-09-27

**Authors:** Qian Sun, Tao Zhuo, Tianyu Zhao, Cuiji Zhou, Yuanyuan Li, Ying Wang, Dawei Li, Jialin Yu, Chenggui Han

**Affiliations:** 1 College of Plant Protection, Shenyang Agricultural University, Shenyang 110866, China; sunqian5328@syau.edu.cn; 2 State Key Laboratory for Agro-Biotechnology and Ministry of Agriculture Key Laboratory of Pest Monitoring and Green Management, College of Plant Protection, China Agricultural University, Beijing 100193, China; zhuotaotao@gmail.com (T.Z.); zhaotianyu@alu.cau.edu.cn (T.Z.); zhoucuiji@fosu.edu.cn (C.Z.); hunan_lyy@cau.edu.cn (Y.L.); yingwang@cau.edu.cn (Y.W.); Dawei.Li@cau.edu.cn (D.L.); yjl@cau.edu.cn (J.Y.)

**Keywords:** PMCV, VSR, F-box protein, SKP1, AGO1

## Abstract

To counteract host antiviral RNA silencing, plant viruses encode numerous viral suppressors of RNA silencing (VSRs). P0 proteins have been identified as VSRs in many poleroviruses. However, their suppressor function has not been fully characterized. Here, we investigated the function of P0 from pea mild chlorosis virus (PMCV) in the suppression of local and systemic RNA silencing via green fluorescent protein (GFP) co-infiltration assays in wild-type and GFP-transgenic *Nicotiana benthamiana* (line 16c). Amino acid deletion analysis showed that N-terminal residues Asn 2 and Val 3, but not the C-terminus residues from 230–270 aa, were necessary for PMCV P0 (P0^PM^) VSR activity. P0^PM^ acted as an F-box protein, and triple LPP mutation (62LPxx79P) at the F-box-like motif abolished its VSR activity. In addition, P0^PM^ failed to interact with S-phase kinase-associated protein 1 (SKP1), which was consistent with previous findings of P0 from potato leafroll virus. These data further support the notion that VSR activity of P0 is independent of P0–SKP1 interaction. Furthermore, we examined the effect of P0^PM^ on ARGONAUTE1 (AGO1) protein stability, and co-expression analysis showed that P0^PM^ triggered AGO1 degradation. Taken together, our findings suggest that P0^PM^ promotes degradation of AGO1 to suppress RNA silencing independent of SKP1 interaction.

## 1. Introduction

RNA silencing is an antiviral immune mechanism in a variety of eukaryotes including fungi, plants, and invertebrates. Antiviral RNA silencing is initiated when virus double-stranded RNA (dsRNA) structures are recognized and are processed into virus-derived small interfering RNAs (vsiRNAs) by RNaseIII-like enzymes Dicer-like proteins (DCLs) [1,2]. Then, vsiRNAs are loaded into ARGONAUTE (AGO)-containing protein complexes called RNA-induced silencing complexes (RISCs) to target viral RNAs for degradation [3,4,5,6,7]. Fragments generated by RISC cleavage are converted into dsRNA by RNA-dependent RNA polymerases (RDRs) and its cofactor SUPPRESSOR OF GENE SILENCING 3 (SGS3) encoded by the plant host to produce secondary vsiRNAs [8,9,10,11]. RDR1, RDR2, and RDR6 are important factors in vsiRNA production during viral infections [10,12,13,14].

To counteract host antiviral RNA silencing, plant viruses have evolved numerous viral suppressors of RNA silencing (VSRs). Although VSRs have been identified in almost all plant virus genera, there is clear diversity in VSR sequence and structure, indicating that they have evolved independently [15]. Recent data have shown that VSRs use various strategies to interfere with different phases of the silencing pathway including initiation of RNA silencing, dicing of viral dsRNA, assembly of RISCs, and amplification by RDRs [16,17,18,19]. A common strategy used by VSRs is to bind dsRNA or siRNA. The turnip crinkle virus (TCV) P38 protein and cucumber mosaic virus (CMV) 2b protein have been found to bind long dsRNA and block vsiRNA biogenesis [2,20,21]. Moreover, tomato bushy stunt virus (TBSV) P19 protein specifically binds to siRNA to prevent siRNA–AGO complex formation [22,23,24]. Several studies have shown that VSRs directly interact with RNA silencing components. V2 from tomato yellow leaf curl virus (TYLCV) inhibits RDR6-mediated amplification by directly interacting with SGS3 [25] and P6 from cauliflower mosaic virus (CaMV), which effectively suppresses silencing by interacting with dsRNA-binding protein 4 (DRB4) [26]. CMV 2b is the first to be identified to interact with AGO1, which inhibits the cleavage activity of AGO1 [27]. TCV P38 directly interacts with and suppresses AGO proteins by mimicking the endogenous GW/WG repetitive motif [28]. In addition, some VSRs have been shown to mediate the degradation of AGO proteins. For example, P25 from potato virus X (PVX) interacts with AGO1, AGO2, AGO3, and AGO4 and selectively promotes degradation of AGO1 in a proteasome-dependent manner [29]. Similarly, CP from tomato ringspot virus (TRSV) binds to and enhances AGO1 degradation through autophagy [30]. P0 proteins, the VSRs of poleroviruses, have also been shown to trigger the degradation of AGO1 via autophagy [31,32,33,34,35].

The poleroviruses are distributed worldwide, infecting many crops of economic importance and causing severe plant diseases and yield losses. As previously reported, P0 proteins of several poleroviruses suppress RNA silencing [33,35,36,37,38,39,40,41,42,43,44,45,46,47]. Although P0 proteins have a very low amino acid sequence identity, they have a conserved F-box-like motif [LPxx(L/I)x_10–13_P] in the N-terminal region and conserved residues [(K/R)IYGEDGX_3_FWR] in the C-terminal region [35,40]. Silencing suppression activity assays indicated that some residues in F-box-like and FWR motifs are important for P0 suppressor activity [32,35,39,40,42,43,44,48]. Other studies have shown that P0 proteins interact via the F-box motif with S-phase kinase-associated protein 1 (SKP1), which belongs to the SKP1–Cullin 1–F-box (SCF) E3 ubiquitin ligase family [35,43,49]. However, AGO1 degradation triggered by P0 is sensitive to autophagy inhibitors rather than proteasome inhibitors [32,34].

In previous research, we identified a tentative novel virus, pea mild chlorosis virus (PMCV), infecting field peas and faba beans in China [50]. PMCV has a genomic organization that is typical of poleroviruses, and the open reading frame ORF0 (nt 51-863) encodes the P0 protein (P0^PM^) [50]. However, whether P0^PM^ protein is a VSR remains unclear. In this study, we investigated the ability of P0^PM^ to suppress local and systemic RNA silencing, trigger AGO1 degradation, and interact with SKP1. In addition, we also identified the critical amino acids responsible for P0^PM^ VSR activity.

## 2. Results

### 2.1. P0^PM^ Suppressed Local but Not Systemic RNA Silencing

**Figure 1:**
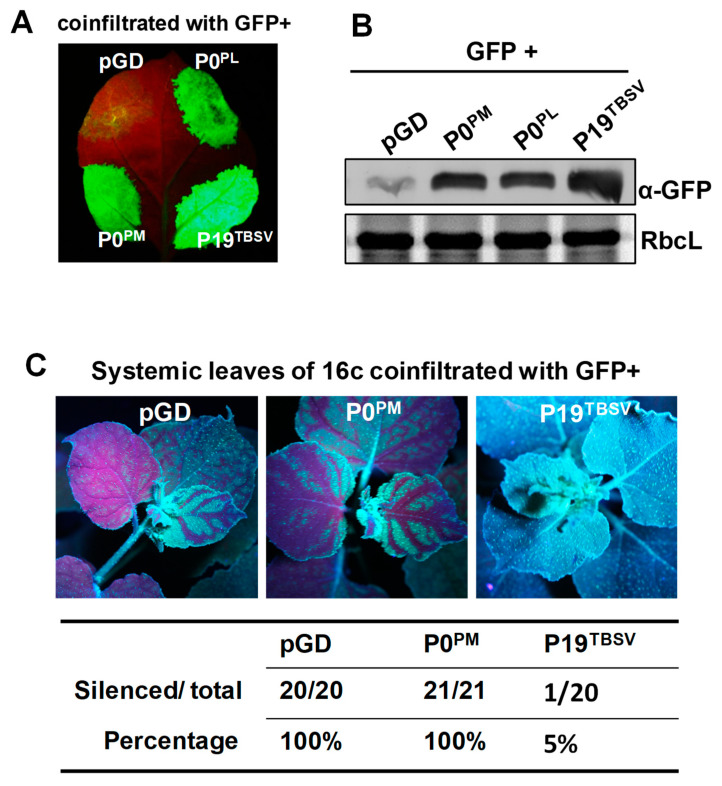
P0^PM^ protein suppressed local but not systemic RNA silencing. (**A**) Agroinfiltration of *N. benthamiana* leaves with green fluorescent protein (GFP) plus empty vector pGD, P0^PL^, P19^TBSV^, or P0^PM^. The empty vector pGD was used as negative control. P0^PL^ and P19^TBSV^ were used as positive controls. Photographs were taken under long-wavelength UV light at 5 days post-infiltration (dpi). (**B**) GFP proteins from infiltrated leaf patches were detected by Western blotting. GFP was detected with GFP polyclonal antiserum. RbcL is the Rubisco large subunit. (**C**) Systemic RNA silencing suppression activity of P0^PM^. GFP was transiently co-expressed in leaves of GFP transgenic *N. benthamiana* 16c plants with pGD, P0^PM^, or P19^TBSV^. The empty vector pGD and P19^TBSV^ were used as negative and positive controls, respectively. Photographs of the upper leaves were taken under long-wavelength UV light at 14 dpi. The ratios below the images show the number of systemic silencing plants out of the total number of infiltrated plants. The systemic silencing efficiency was scored in three independent experiments.

To test whether P0^PM^ is an RNA silencing suppressor, a green fluorescent protein (GFP) agroinfiltration assay was performed in *Nicotiana benthamiana* [51,52]. *Agrobacterium tumefaciens* harboring the binary vector pGDG that expressed GFP [53] was mixed with *A. tumefaciens* containing binary constructs coding for P0^PM^, potato leafroll virus (PLRV) P0 (P0^PL^), TBSV P19 (P19^TBSV^), or empty binary vector pGD as described in the methods. The empty vector pGD served as a negative control, and P0^PL^ and P19^TBSV^ were used as positive controls [43,54]. All the mixtures were transiently co-infiltrated into *N. benthamiana* leaves. Leaf patches infiltrated with GFP/pGD showed negligible GFP fluorescence under long-wavelength UV light at 5 days post-infiltration (dpi), which indicated effective induction of GFP RNA silencing (Figure 1A). In contrast, leaf patches infiltrated with GFP/P0^PM^ revealed strong GFP fluorescence similar to GFP/P0^PL^ and GFP/P19^TBSV^. To confirm these observations, the GFP protein level was measured by Western blot, and we found that GFP protein levels were consistent with the results described above and were rarely detected in the leaf patches infiltrated with GFP/pGD, while GFP/P0^PM^-infiltrated leaf patches showed high accumulation of GFP similar to the GFP/P0^PL^ and GFP/P19^TBSV^ positive controls (Figure 1B). These results indicate that P0^PM^ was able to suppress local GFP RNA silencing.

We conducted GFP agroinfiltration assays in *N. benthamiana* 16c [52] to analyze whether P0^PM^ suppresses the long-distance spread of the systemic silencing signal. At 14 dpi, GFP fluorescence signals were observed in the upper leaves under long-wavelength UV light, and the percentage of systemic silencing suppression was calculated (Figure 1C). All plants infiltrated with GFP/pGD or GFP/P0^PM^ exhibited systemic RNA silencing. In contrast, systemic RNA silencing was suppressed in plants infiltrated with GFP/P19^TBSV^ with only 5% exhibiting systemic RNA silencing (Figure 1C). These results indicate that P0^PM^ did not suppress systemic RNA silencing.

### 2.2. Requirement of N- and C-Terminal Amino Acids of P0^PM^ for Suppressor Activity

**Figure 2:**
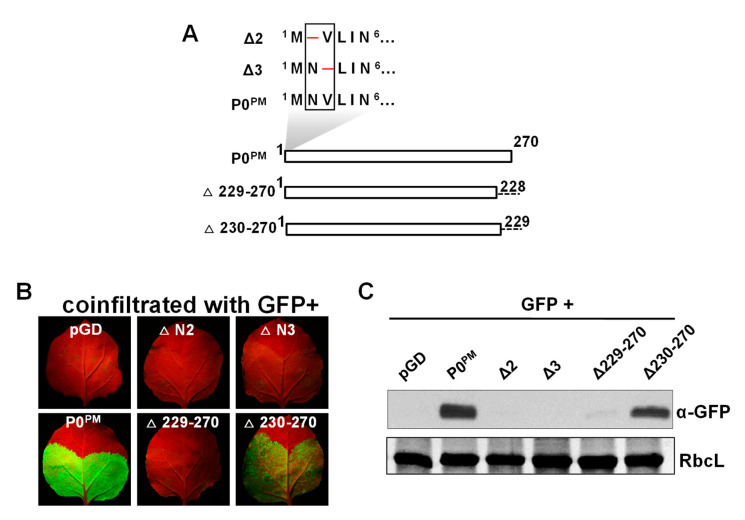
Requirement of N- and C-terminal amino acids of P0^PM^ for suppressor activity. (**A**) Schematic representation of amino acids deleted (─) in the N-terminus of P0^PM^ (upper panel) and C-terminal truncation mutants (lower panel). Numbers correspond to the amino acid positions within the P0^PM^ sequence. (**B**) Agroinfiltration of *N. benthamiana* leaves with GFP plus empty vector pGD, P0^PM^ mutants or P0^PM^, respectively. The empty vector pGD was used as negative control. Photographs were taken under long-wavelength UV light at 5 dpi. (**C**) GFP proteins from infiltrated leaves were detected by Western blotting at 5 dpi. GFP was detected with GFP polyclonal antiserum. RbcL is the Rubisco large subunit.

According to the previous studies, the N- and C-terminal amino acids are critical for silencing the suppressor activity of P0 [39,46]. To verify whether the N- and C-terminal amino acids are required for P0^PM^ VSR activity, N-terminal deletion mutants Δ2 and Δ3 representing a single amino acid deletion at position 2 or 3, respectively, and C-terminal truncation mutants Δ229–270 and Δ230–270 representing deletion of 42 or 41 amino acids in the C-terminus, respectively, were constructed as described in the methods (Figure 2A). Then, wild-type *N. benthamiana* were co-infiltrated with *A. tumefaciens* carrying pGDG and *A. tumefaciens* carrying these four P0^PM^ mutants, wild-type P0^PM^, or empty binary vector pGD. At 5 dpi, GFP fluorescence in leaves co-infiltrated with GFP and Δ2, Δ3, or Δ229-270 was not detected as in leaves co-infiltrated with GFP/pGD (Figure 2B). Interestingly, the leaves co-infiltrated with GFP/Δ230-270 showed a high level of GFP fluorescence similar to GFP/P0^PM^ (Fig. 2B). Western blot analyses showed that GFP protein accumulation was consistent with GFP fluorescence levels (Figure 2C). These results indicate that the N-terminal residues Asn 2 and Val 3 of P0^PM^ were necessary for RNA silencing suppression function, whereas C-terminal 41 residues (230–270 aa) were not required.

### 2.3. Critical Residues in P0^PM^ Motifs Were Required for Suppressor Activity

**Figure 3:**
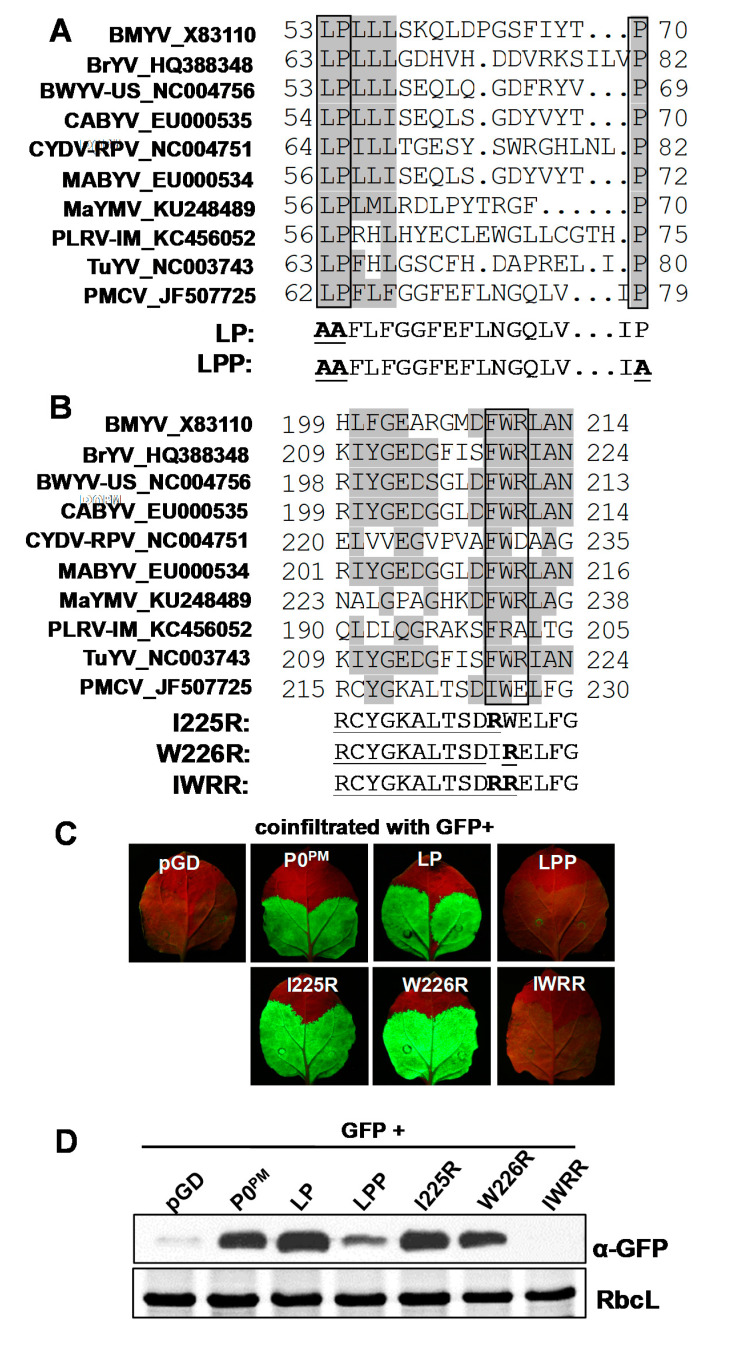
The critical residues in P0^PM^ motifs were required for its suppressor activity. (**A**) Multiple alignment of the F-box-like domains of P0 amino acid sequences from 10 poleroviruses. The region (amino acids 62 to 79) in P0^PM^ has homology to F-box-like domains. The LP mutant of P0^PM^ indicates a double mutation at amino acids 62 and 63 from LP to AA. The LPP mutant of P0^PM^ indicates a triple mutation at amino acids 62, 63, and 79 from LPP to AAA. (**B**) Multiple alignment of the FWR motif of P0 amino acid sequences from 10 poleroviruses. I225R mutant of P0^PM^ indicates the point mutation at amino acid 225 from I to R. W226R mutant of P0^PM^ indicates the point mutation at amino acid 226 from W to R. The IWRR mutant of P0^PM^ indicates a double mutation at amino acids 225 and 226 from IW to RR. (**C**) Agroinfiltration of *N. benthamiana* leaves with GFP plus empty vector pGD, LP, LPP, I225R, W226R, IWRR, or P0^PM^. The empty vector pGD was used as negative control. Photographs were taken under long-wavelength UV light at 5 dpi. (**D**) GFP proteins from infiltrated leaf patches were detected by Western blotting at 5 dpi. GFP was detected with GFP polyclonal antiserum. RbcL is the Rubisco large subunit.

The above results showed that P0^PM^ is an RNA silencing suppressor; thus, we determined whether it also functions as an F-box-like protein. Multiple alignment of the P0 amino acid sequences from 10 poleroviruses revealed that P0^PM^ had a similar sequence (**LP**FLFGGFEFLNGQLVI**P**) to the F-box-like motif [LPxx(L/I)X_10–13_P] from residues 62 to 79 (Figure 3A). Interestingly, residue 66 at the L/I position was an F instead of the consensus L or I residue. To determine whether the similar F-box-like motif in P0^PM^ is functional for P0^PM^ VSR activity, a series of P0^PM^ mutants in the F-box-like motif was constructed including LP (amino acids 62 and 63 from LP to AA) and LPP (amino acids 62, 63, and 79 from LPP to AAA). Then, LP or LPP mutants were transiently co-infiltrated with GFP in *N. benthamiana*. At 5 dpi, GFP fluorescence showed that the LP mutant did not affect its VSR activity; however, the LPP mutant abolished its VSR activity (Figure 3C). These results were consistent with a previous observation for enamovirus pea enation mosaic virus-1 P0, where substitution of the conserved LP residues did not affect the suppressor activity while substitution of the conserved LPP residues inhibited its suppressor activity [55]. Western blot analyses showed that GFP protein accumulation was consistent with fluorescence levels (Figure 3D). These results demonstrate that the similar F-box-like domain was essential for P0^PM^ suppressor function.

Most P0 proteins have a conserved C-terminal-proximal sequence (K/R) IYGEDGX_3_FWR, and it has been demonstrated that the residues present in (K/R) IYGEDGX_3_FWR affect P0 silencing suppressor activity [40,43,46]. Although the C-terminal-proximal sequence (RCYGKALTSDIWE) of P0^PM^ displayed only 40% identity with the consensus sequence (Figure 3B), we found that the P0^PM^ mutant IWRR (amino acids 225 and 226 from IW to RR) destroyed P0^PM^ VSR activity. Mutation of amino acid 225 or 226 (I225 and W226, respectively) alone also suppressed RNA silencing (Figure 3C). Western blot analyses showed that GFP protein accumulation was consistent with GFP fluorescence levels (Figure 3D). These results indicate that I225/W226 residues were essential for P0^PM^ suppressor activity.

### 2.4. P0^PM^ Failed to Interact with SKP1 in Yeast

**Figure 4:**
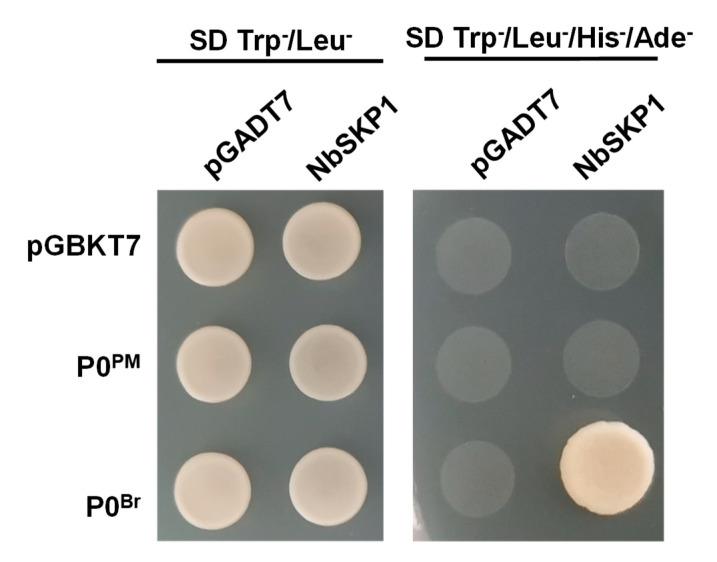
P0^PM^ protein failed to interact with NbSKP1. Analysis of interactions between P0^PM^ and NbSKP1 using the yeast two-hybrid system. P0^PM^ and P0^Br^ were cloned into bait vector pGBKT7 and transformed into yeast Y187, respectively. NbSKP1 was cloned into prey vector pGADT7 and transformed into yeast AH109. The interaction of P0^Br^ and NbSKP1 was used as a positive control. Yeast strains were grown on SD/-Leu/-Trp and SD/-Ade/-His/-Leu/-Trp and maintained at 30 °C for 3–5 days.

Previous studies have shown that polerovirus P0 proteins interact via the F-box-like motif with SKP1, which is a core component of SCF E3 ubiquitin ligase complex [35,46,49]. However, not all F-box P0 proteins interact with SKP1 such as P0^PL-IM^ [43]. As shown above, P0^PM^ has a similar F-box like motif; therefore, we examined the interaction between P0^PM^ and SKP1 using a yeast two-hybrid (Y2H) system. The ORFs of P0^PM^ and *N. benthamiana* SKP1 (NbSKP1) were cloned into the GAL4 DNA binding domain (BD) and GAL4 activation domain (AD), respectively. The interaction of P0 from brassica yellows virus P0 (P0^Br^) and NbSKP1 was used as a positive control [46]. Different combinations were mixed for mating, and the mixed transformants were transferred to synthetic dropout (SD) media lacking leucine and tryptophan (SD/–Leu/–Trp) and SD media lacking leucine, tryptophan, adenine, and histidine (SD/–Leu/–Trp/–Ade/–His) plates for 3−5 days. All mixed transformants were able to grow on SD/–Leu/–Trp plates; however, only yeast transformed with BD-P0^Br^ and AD-NbSKP1 grew on SD/–Leu/–Trp/–Ade/–His plates (Figure 4). Thus, P0^PM^ did not interact with NbSKP1 in yeast.

### 2.5. AGO1 Protein Destabilization Triggered by P0^PM^ Was Correlated with Suppressor Activity

**Figure 5:**
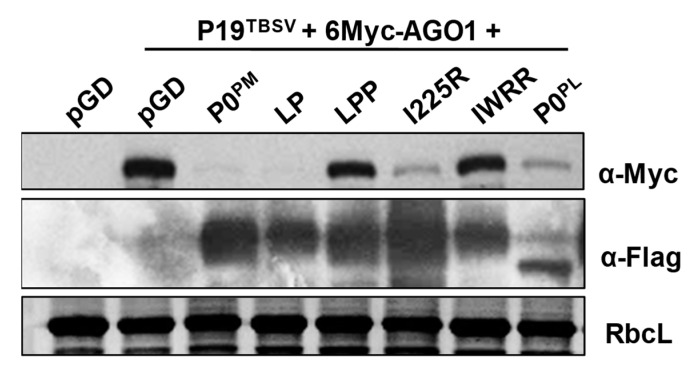
Effect of P0^PM^ and its mutants on the accumulation of AGO1 protein. The 6Myc-tagged AtAGO1 was transiently co-expressed with 3Flag-tagged P0^PM^ and its mutants in *N. benthamiana* leaves through agroinfiltration in the presence of P19^TBSV^. Protein samples were prepared from plant leaves at 5 dpi. The accumulation of AtAGO1 and P0^PM^ and its mutants was detected by Western blotting. AtAGO1 was detected with c-Myc monoclonal antibody; P0^PM^ and its mutants were detected with Flag monoclonal antibody. RbcL is the Rubisco large subunit.

Previous studies showed that poleroviruses’ P0s target AGO1 protein for degradation [31,32,43,46]. Therefore, we investigated whether P0^PM^ could also degrade AGO1 protein. To determine the effect of P0^PM^ on AGO1 protein stability, we performed an agroinfiltration assay. The 6×Myc-tagged *Arobidopsis* AGO1 (6Myc-AtAGO1) was transiently co-expressed with 3×Flag-tagged P0^PM^ or P0^PL^ in *N. benthamiana* leaves through agroinfiltration. P0^PL^-induced AtAGO1 degradation was used as a positive control [43]. P19^TBSV^ was added in this assay to ensure expression of 6Myc-AtAGO1, and protein samples were analyzed at 5 dpi. As shown in Figure 5, the level of 6Myc-AtAGO1 was significantly reduced in the presence of P0^PL^ compared with the empty vector pGD. When 6Myc-AtAGO1 was co-expressed with P0^PM^, 6Myc-AtAGO1 protein was hardly detected, indicating that P0^PM^ triggered AGO1 degradation.

To further test whether AGO1 degradation is correlated with P0^PM^ VSR activity, the P0^PM^ mutants LP, LPP, I225R, and IWRR were transiently co-expressed with 6Myc- AtAGO1. Western blot analysis of 6Myc-AtAGO1 protein revealed that LP and I225R with VSR activity were able to trigger AGO1 degradation, whereas LPP and IWRR, without VSR activity, did not affect AGO1 accumulation, which was similar to the empty vector pGD (Figure 5). Taken together, these results suggest that P0^PM^ suppressor activity was correlated with AGO1 degradation.

## 3. Discussion

In this study, we investigated the function of the P0 protein encoded by the first ORF of PMCV. Like previously studied polerovirus P0 proteins, P0^PM^ is an RNA silencing suppressor with various levels of activity [33,35,36,37,38,39,40,41,42,43,44,45,46]. At present, P0 proteins from potato leafroll virus (PLRV), turnip yellows virus (TuYV, synonyms beet western yellows virus FL strain, BWYV-FL), beet mild yellowing virus (BMYV), cucurbit aphid-borne yellows virus (CABYV), cotton leafroll dwarf virus (CLRDV), melon aphid-borne yellow virus (MABYV), maize yellow dwarf virus-RMV2 (MYDV-RMV2), brassica yellows virus (BrYV), sugarcane yellow leaf virus (ScYLV), maize yellow mosaic virus (MaYMV), and cereal yellow dwarf virus (CYDV) suppress local RNA silencing [36,39,40,41,42,43,44,45,46,47,49,55]. Among them, the P0 proteins of PLRV, CYDV, ScYLV, MaYMV, BrYV, and MYDV-RMV2 also suppress systemic RNA silencing, in contrast to the P0 proteins of CLRDV, TuYV, and CABYV. Whether systemic suppression is induced by the P0 proteins of MABYV and BMYV is not reported [40,41]. In addition, the P0 proteins from TuYV, ScYLV, PLRV, BrYV, CABYV, and CYDV trigger cell death within the infiltration patch in *Nicotiana* species [33,39,48,56], and the P0 proteins of ScYLV and TuYV trigger dose-dependent cell death in infiltrated *N. benthamiana* leaves. Recent studies show that P0^Tu^, P0^PL^, and P0^CA^ elicit a hypersensitive response in *N. glutinosa*, and P0^Tu^ is recognized by a resistance gene. In our study, we found that P0^PM^ suppressed local RNA silencing but not systemic RNA silencing in an *A. tumefaciens*-mediated co-infiltration assay in wild-type and GFP-transgenic *N. benthamiana*. However, we did not observe any occurrences of cell death in the P0^PM^-inoculated *N. benthamiana* leaves. The reason for the differences in local and systemic RNA silencing suppression activity and hypersensitive responses between these P0s remains unclear.

Recently, we demonstrated that BrYV-A P0 (P0^BrA^) is a VSR that suppresses both local and systemic RNA silencing [46]. Deletion of a single amino acid at position 2 or 3 in the N-terminus of P0^BrA^ abolishes its local and systemic RNA silencing suppression, while deletion of 25 amino acid residues in the C-terminus (225–249 aa) still allows for suppression of local RNA silencing but not systemic RNA silencing [46]. In addition, studies show that deletion of 15 N-terminal amino acid residues (2–15 aa) of SCYLV P0 abolishes both local and systemic RNA silencing suppression activity. In contrast, only systemic RNA silencing suppression was abolished when the 15 C-terminal amino acid residues were deleted [39]. In this study, we found that single amino acid deletion at position 2 or 3 in the N-terminus of P0^PM^ abolished its VSR activity, and deletion of 41 C-terminal amino acid residues (230–270 aa) did not have any effect. This could be explained by our prediction of the secondary structure of P0^PM^, in which we found that the C-terminal domain did not form any structure. Taken together, these results indicate that N-terminal residues in the P0 proteins are essential for VSR activity, but C-terminal residues are not required for its local VSR activity; only those P0 with systemic VSR activity need its C-terminal residues. The reason for the difference in systemic RNA silencing suppression between these P0s and role of the C-terminal residues should be further investigated. Thus, obtaining and analyzing the crystal structure of P0 may provide more explanation as to how these amino acids affect the suppression of RNA silencing.

Multiple alignment of the P0 proteins from poleroviruses revealed that P0^PM^ had a similar F-box-like motif. Substitution of the conserved LPP residues in the F-box-like motif of P0^PM^ abolished suppressor activity, which was consistent with a previous publication [48]. Our results indicated that P0^PM^ also functioned as an F-box protein. In addition, we found that substitution of the C-terminal I225/W226 residues destroyed P0^PM^ suppressor activity. Early studies have shown that P0 proteins interact with SKP1 via its F-box-like motif [35,46,49]. Point mutations in the F-box-like motif of P0 not only abolish the silencing suppressor activity of P0 but also abolish the P0–SKP1 interaction [35]. Our Y2H analysis revealed that F-box protein P0^PM^ failed to interact with NbSKP1. Interestingly, we discovered that residue 66 of P0^PM^ at the L/I position of F-box-like motif was an F instead of the consensus L or I residue, which may be the reason why P0^PM^ was unable to interact with NbSKP1. Additional methods for investigating P0^PM^–SKP1 interaction should be performed in future studies. Studies have demonstrated that not all F-box P0 proteins interact with SKP1. For example, P0^PL^ also has a conserved F-box-like motif and suppresses RNA silencing but fails to interact with SKP1 [43]. In addition, a recent study of BrYV P0 showed that the P0^Br^–SKP1 interaction is not directly required for its suppression activity but instead is needed for protecting P0^Br^ from degradation through autophagy and proteasome pathways [46]. Our data further supported this finding that RNA silencing suppression activity of P0 is independent of P0–SKP1 interaction. However, it is likely that P0^PL^ and P0^PM^ may interact with SKP1 from natural hosts or other SKP1-like proteins from *N. benthamiana* that have same activity with SKP1.

Suppression of RNA silencing by P0 is associated with degradation of AGO1 protein [31,32]. We demonstrated that P0^PM^ triggered AGO1 degradation and LP and I225R mutants did affect AGO1 accumulation, whereas LPP and IWRR had no effect on AGO1 accumulation and consequently were unable to suppress RNA silencing. Furthermore, we found that AGO1 degradation was correlated with P0^PM^ suppressor activity. Previous research has shown that P0^Tu^ and P0^CA^ directly bind to AGO1, although others like WYDV-RMV2 P0 fail to interact with AGO1 [32,45]. Whether P0^PM^ directly interact with AGO1 and/or other proteins of the RNA silencing pathway to promote AGO1 degradation or indirectly influence AGO1 stability by targeting another protein capable of stabilizing AGO1 still needs to be elucidated in future studies.

## 4. Materials and Methods 

### 4.1. Plant Material and Growth Conditions

Wild-type Nicotiana benthamiana and green fluorescent protein (GFP) transgenic N. benthamiana 16c plants were grown at 24 °C with a 16 h light/8 h dark cycle.

### 4.2. Plasmid Constructs

All of the primers are listed in Table 1.

The vectors pGD and pGDG [53] were used for transient expression. For the VSR activity assay, P0^PM^ and its mutants with stop codon were cloned into pGD for the GFP agroinfiltration assay. In the AGO1 degradation experiment, P0^PM^ and corresponding P0^PM^ mutants without stop codon were cloned into pGD–3Flag, a modified version of vector pGD that has a C-terminal-fused 3×Flag tag.

For the Y2H assay, the full length of P0^PM^ was cloned into pGDBKT7 containing a binding domain (BD) to generate BD–P0^PM^. Construction of pGDBKT7–P0^BrA^ and pGAD–NbSKP1 was described previously [46,57].

### 4.3. Agrobacterium-Mediated Transient Expression and GFP Imaging

Plasmids were transformed into the *Agrobacterium tumefaciens* strain EHA105 using the freeze–thaw method [58]. Co-infiltration assays were performed as previously described [59]. The recombinant EHA105 or C58CI was grown overnight; resuspended in infiltration buffer (10 mM MgCl_2_, 10 mM MES, and 100 μM acetosyringone); and incubated at room temperature for at least 3 h before infiltration. The *A. tumefaciens* cultures were infiltrated into *N. benthamiana* leaves, and then the infiltrated leaves were detached for the corresponding assays. Final dilutions of the cultures used in co-infiltration assays were 0.5 OD600 for each construct.

Leaves were illuminated under a BLAK-RAY non-UV semiconductor inspection lamp (B-100AP/R; UVP Inc., Upland, CA, USA), and images were taken with a digital camera (CoolPix 4500; Nikon, Tokyo, Japan) at 5 and 14 days post-infiltration (dpi). All experiments shown in the results were repeated at least three times.

### 4.4. Yeast Two-Hybrid (Y2H) Assay

For the Y2H assay, the Clontech Matchmaker GAL4 Two-Hybrid System 3 (Clontech, Mountain View, CA, USA) was used. BD, BD–P0^PM^, and BD–P0^BrA^ were transformed into the yeast host strain Y187. AD and AD–NbSKP1 were transformed into the yeast host strain AH109. Protein interactions were tested using the yeast mating assay. The mated transformants were plated onto synthetic dropout (SD) media lacking Trp and Leu (SD/−WL) and SD media lacking Ade, His, Trp, and Leu (SD/−AHLW). The plates were incubated at 30 °C for 3–5 days.

### 4.5. Protein Extraction and Western Blots

Total proteins were extracted from infiltrated *N. benthamiana* leaves using 2× sodium dodecyl sulfate (SDS) sample buffer (100 mM Tris, pH 6.8; 4% *w/v* SDS; 20% *v/v* glycerol; and 0.2% *w/v* bromophenol blue). Proteins were separated with SDS polyacrylamide gel electrophoresis. Western blots were performed with the primary anti-Flag antibody (1:1,000; Sigma-Aldrich, St. Louis, MO, USA), anti-GFP antibody (1:3000), or anti-anti-c-myc antibody (1:1000; Sigma-Aldrich, St. Louis, MO, USA) and then incubated with anti-rabbit goat HRP secondary antibody (1:3000; Bio-Rad, Hercules, CA, USA). Finally, the membrane was detected with an enhanced chemiluminescence detection kit (GE Healthcare, Buckinghamshire, UK) according to the manufacturer’s instructions. 

## Figures and Tables

**Table 1:** Primer sequences used for polymerase chain reaction.

**Primer**	**Sequence (5′–3′)**
P0XhoF	TATCTCGAGATGAACGTGTTAATC
P0TGA-ApaR	TATGGGCCCTCATAGCTCCCAAAACCCTTCG
P0ApaR	TATGGGCCCTAGCTCCCAAAACCCTTCG
Δ2XhoF	TATCTCGAGATGGTGTTAATCAATCAATACAC
Δ3XhoF	TATCTCGAGATGAACTTAATCAATCAAT
Δ229-270TGAR	CGGGATCCGGGCCCTCAAAGCTCCCAAATATCAGATG
Δ229-270-R	TATGGGCCCAAGCTCCCAA ATATCAGATG
**Primer**	**Sequence (5′-3′)**
Δ230-270TGAR	CGGGATCCGGGCCCTCAGAAAAGCTCCCAAATATCAG
Δ230-270-R	TATGGGCCCGAAAAGCTCC CAAATATCAG
LP-F	GCCGCTTTTCTATTCGGGGGCTTC
LP-R	CAAAAGCAGAAGAGAGCGATAGC
LPP-F	TTACACGGGAGTAACCG
LPP-R	GCGGGCGATGACCAGTTG
I225R-F	GATAGGTGGGAGCTTTTCGGT
I225R-R	AGATGTAAGAGCTTTGCCGTAG
W226R-F	GATATTAGGGAGCTTTTCGGT
P0NdeF	TATCATATGAACGTGTTAATC
P0BamHR	TATGGATCCTCATAGCTCCCAAAACCCTTCG

## Abbreviations

AGO	ARGONAUTE
BMYV	Beet mild yellowing virus
BrYV	Brassica yellows virus
CABYV	Cucurbit aphid-borne yellows virus
CLRDV	Cotton leafroll dwarf virus
CYDV	Cereal yellow dwarf virus
MABYV	Melon aphid-borne yellow virus
MaYMV	Maize yellow mosaic virus
MYDV	Maize yellow dwarf virus
PMCV	Pea mild chlorosis virus
PLRV	Potato leafroll virus
ScYLV	Sugarcane yellow leaf virus
TBSV	Tomato bushy stunt virus
VSRs	Viral suppressors of RNA silencing
